# Lipid layer thickness decrease due to meibomian gland dysfunction leads to tear film instability and reflex tear secretion

**DOI:** 10.1080/07853890.2022.2056238

**Published:** 2022-04-05

**Authors:** Wung Jae Kim, Ye Jin Ahn, Min Ho Kim, Hyun Seung Kim, Man Soo Kim, Eun Chul Kim

**Affiliations:** a Gwangjin St. Mary’s Eye Clinic, Seoul, Republic of Korea; b Department of Ophthalmology, College of Medicine, The Catholic University of Korea, Seoul, Republic of Korea

**Keywords:** Lipid layer thickness, Lipiview interferometer, Meibomian gland dysfunction, aqueous deficient dry eye, tear film instability

## Abstract

**Purpose:**

To determine the clinical effects of ocular surface and Meibomian gland parameters on tear film stability among individuals with Meibomian gland dysfunction (MGD), those with aqueous deficient dry eye (ADDE), individuals with both conditions and normal controls.

**Methods:**

Patients were divided into four groups: normal controls, patients with ADDE, patients with MGD, and patients who fulfilled diagnostic criteria for ADDE and MGD (Mixed Group). Data for ocular symptom score, lid margin abnormality, ocular staining, tear break-up time, meiboscore, and lipid layer thickness (LLT) measured by a Lipiview interferometer, Schirmer test, and MGD severity score were collected.

**Results:**

A total of 109 patients (109 eyes) were evaluated. In patients with MGD, LLT was significantly lower than the ADDE patients. However, the Schirmer test value was the highest in the MGD group. The LLT negatively correlated with meiboscore and MGD severity score in the MGD group. Significant correlation between Schirmer test value and meiboscore was definite in the MGD group.

**Conclusions:**

Tear fluid secretion is more increased and lipid layer thickness is more decreased in MGD patients than in ADDE patients. Decreased lipid layer thickness caused by MGD-related tear film instability may stimulate reflex tear secretion. The obstructive MGD is more prevalent than hypersecretary MGD.

**Key messages**
The tear film stability is affected by Mebomian gland dysfunction (MGD). The measurement of the tear film parameters including lipid layer thickness suggests that the obstructive MGD is more prevalent than hypersecretary MGD and the aqueous layer compensates the decreased lipid layer caused by MGD.

## Introduction

According to the International Dry Eye Work Shop (DEWS), dry eye is a disease of the tear and ocular surfaces causing symptoms of discomfort, visual disturbance, and tear film instability [1]. The normal functioning ocular surface operates by homeostasis of the stable and sufficient tear film [2]. The role of the lipid layer is to stabilize the tear film and prevent tear evaporation from the aqueous tear film layer. However, this homeostasis may be affected by Meibomian gland dysfunction (MGD), which is characterized by terminal duct obstruction and changes in glandular secretion [3]. MGD is associated with multiple pathophysiological mechanisms including eyelid inflammation, conjunctival inflammation, corneal damage, microbiological changes, and dry eye [4]. It is now widely accepted that MGD is divided into two major categories based on Meibomian gland secretion: first is the hyposecretory or obstructive subtype and the second is the hypersecretory subtype [5]. Both of these conditions may cause the instability of the tear film, however, there is lack of data concerning the correlation of clinical parameters with tear film homeostasis in MGD.

Arita et al. [6] previously supposed that the lipid layer and aqueous layer compensate each other to maintain homeostasis. The aim of this study was to investigate the relationships between Meibomian gland dysfunction and the tear film parameters including lipid layer thickness (LLT) among individuals with MGD, those with aqueous deficient dry eye (ADDE), those with both conditions, and in normal controls.

## Materials and methods

### Methods

This study was performed retrospectively through chart review, and the need of the written informed consent has waived by the Institutional Review Board of Bucheon St. Mary Hospital, which approved this study (HC11RIMI000).

### Subjects

This study comprises women older than 40 years who visited Bucheon St. Mary’s Hospital, The Catholic University of Korea between January 2016 and September 2016, reporting dry eye symptoms. Patients with systemic diseases, ocular diseases except cataract, and past history of ocular surgery or punctual plugging were excluded. Those who were taking oral medication or had been applying topical eye drops with the exception of artificial tears and contact lens wearers were also excluded from the study. Individuals who used artificial tears were instructed not to administer them for at least 2 h prior to the examinations.

### Clinical examinations

Dry eye was diagnosed according to the Korean diagnostic criteria as described elsewhere [7]. The subjects were instructed not to use any eye drops up to two hours before the examinations. The tests for dry eye were performed in the following order: (1) Ocular Surface Disease Index (OSDI) was calculated to evaluate dry eye symptoms (tearing and foreign body sensation), performing ability (reading and driving), and uncomfortable situation (windy and low humidity condition) [8]. (2) Lid margin abnormalities were scored from 0 through 4 according to the findings present. Irregular lid margin, vascular engorgement, plugged Meibomian gland orifices, and anterior or posterior replacement of the mucocutaneous junction were observed by slit-lamp microscopy. (3) Corneal and conjunctival staining was evaluated after fluorescein staining under a yellow-barrier filter and cobalt blue illumination. Corneal and conjunctival staining was graded according to the Sjögren’s International Collaborative Clinical Alliance (SICCA) ocular staining score (OSS) [9]. (4) To measure tear film break-up time (TBUT), a fluorescein-impregnated strip was placed in the lateral part of the inferior fornix, and the patient was asked to blink. The time before the corneal dry spot appeared in the stained tear film was recorded as TBUT. TBUT ≤5 s was considered abnormal [10]. (5) The lower eyelids were evaluated using a Lipiview interferometer (TearScienceInc, Morrisville, NC). The degree of Meibomian gland loss was scored according to the meiboscore (Grade 0: no dropout, Grade 1: dropout of 2/3 of lid area, Grade 2: dropout of 1/3–2/3 of lid area, Grade 3: dropout of >2/3 of lid area) [11]. In addition, LLT was measured as described by Blackie et al. [12]. The LLT is presented in interferometric colour units, in which 1 interferometric colour unit corresponds to approximately 1 nm. An LLT of roughly 70 nm is considered normal. (6) The Schirmer test (without anesthesia) was performed before instillation of any eye drops. The standardized Schirmer test strip (Eagle Vision, Memphis, TN) was bent and placed at the inferior outer fornix. Each patient was instructed to keep his/her eyes closed during the test. The length of maximal wetting was measured after 5 min. A Schirmer test value ≤5 mm was considered abnormal [10]. (7) MGD severity was assessed by clinical parameters and ocular symptom scores, which ranged from 0 through 5 [13]. The examiner assessed presence of an inflamed lid margin. The degree of Meibomian gland expressibility was graded by pressing the five glands of the central third of the lower lid: grade 0, all five glands expressible; grade 1, three to four glands expressible; grade 2, one to two glands expressible; and grade 3, no glands expressible. The meibum quality over the eight lower lid glands was graded as follows: grade 0, clear; grade 1, cloudy; grade 2, cloudy with granular debris; and grade 3, thick, like toothpaste. Each of the eight glands of the lower eyelid was graded on a scale from 0 to 3. The scores of the eight glands were summed to obtain a total score (range, 0–24) [14,15]. To improve the accuracy of the examinations, LLT was measured first and then MGD severity.

All participants were divided into four groups: the normal group comprised subjects who met the following criteria: (1) OSDI less than 12, (2) no tear film abnormality (Schirmer test value ≥5mm and TBUT ≥5 s), and (3) no abnormalities of the lid margins and meibum. The ADDE group included subjects who fulfilled the following criteria: (1) presence of dry eye symptoms (OSDI ≥12), (2) abnormal tear production as determined by the Schirmer test (<5 mm after 5 min) or abnormal tear film stability as determined by TBUT (<5 s), and (3) presence of conjunctival and corneal epithelial damage as evidenced by a fluorescein staining score ≥3, based on the SICCA ocular staining score. Patients with Sjögren’s syndrome were excluded from the study. The MGD group included subjects who fulfilled the following criteria: (1) presence of dry eye symptoms (OSDI ≥12), (2) at least 1 lid margin abnormality, and (3) poor meibum secretion (MGD staging grades 1–5). The ADDE and the MGD group (mixed group) were composed of candidates who met the entry criteria for both the ADDE and MGD groups.

### Statistical analysis

Statistical analysis was performed using SPSS software (version 22.0, SPSS, Inc., SPSS Inc., Chicago, IL). Data for all parameters are presented as mean ± standard deviation. Only one eye from each age-matched female subject was included for analysis. Comparison between the groups was performed using one-way analysis of variance. Regression curves comparing independent samples were calculated on the basis of scatterplots, and the Spearman’s correlation coefficient (*r*) was used for evaluation. A *p*-value less than .05 was considered statistically significant.

## Results

A total of 109 patients (109 eyes) were enrolled in this study. The normal, ADDE, MGD, and Mixed groups consisted of 30 (mean age 53.79 ± 8.75 years), 29 (54.25 ± 11.27 years), 23 (56.95 ± 11.41 years), and 27 (59.45 ± 10.77 years) subjects, respectively. The clinical parameters and the pairwise comparison among the four groups are shown in Tables 1 and 2, respectively.

**Table 1.** Demographic clinical parameters of study subjects.

Parameter	Normal	ADDE	MGD	ADDE/MGD (mixed group)
Total patients	30	29	23	27
Age (years)	53.79 ± 8.75	54.25 ± 11.27	56.95 ± 11.41	59.45 ± 10.77
OSDI score	19.26 ± 8.91	48.65 ± 21.31	47.01 ± 21.39	50.55 ± 25.34
Tear film BUT (seconds)	9.33 ± 1.15	3.26 ± 1.77	2.89 ± 1.52	3.01 ± 1.32
Schirmer I (mm)	10.04 ± 1.75	1.88 ± 2.79	14.02 ± 5.25	3.72 ± 3.01
Fluorescein staining (0–12)	0.16 ± 1.27	3.77 ± 1.16	1.03 ± 1.16	3.57 ± 1.81
Lid margin abnormality (0–4)	0.00 ± 0.00	1.18 ± 0.31	2.31 ± 0.70	2.33 ± 0.43
Meiboscore (0–3)	0.00 ± 0.00	0.77 ± 0.63	1.81 ± 0.63	1.41 ± 0.25
Lipid layer thickness (nm)	88.51 ± 5.72	76.50 ± 21.25	52.25 ± 25.50	57.33 ± 24.41
MGD severity level (0–5) (5: plus disease)	0.00 ± 0.00	0.00 ± 0.00	1.59 ± 0.26	1.30 ± 0.72

ADDE: aqueous deficient dry eye; MGD: meibomian gland dysfunction; OD: right eye; OSDI: ocular surface disease index; BUT: break-up time.

**Table 2.** Statistical comparison of clinical parameters of four groups.

Parameter	Normal versus ADDE	Normal versus MGD	MGD versus ADDE	ADDE versus mixed	MGD versus mixed	Normal versus mixed
Age	0.450	0.725	0.322	0.402	0.258	0.673
OSDI score	0.000*	0.000*	0.875	1.000	0.658	0.001*
Tear film BUT (seconds)	0.000*	0.001*	0.962	1.000	0.733	0.001*
Schirmer I (mm)	0.000*	0.415	0.000*	0.493	0.000*	0.000*
Fluorescein staining (0–9)	0.000*	0.625	0.000*	0.385	0.209	0.000*
Lid margin abnormality (0–4)	0.497	0.031*	0.311	0.145	1.000	0.012*
Meiboscore (0–3)	0.025*	0.001*	0.004*	0.002*	0.577	0.000*
Lipid layer thickness (nm)	0.965	0.011*	0.036*	0.009*	0.795	0.011*
MGD severity level (0–5) (5: plus disease)	–	0.001*	0.001*	0.000*	0.831	0.000*

* *p*-value <.05.

ADDE: aqueous deficient dry eye; MGD: meibomian gland dysfunction; OSDI: ocular surface disease index; BUT: break-up time; mixed group: ADDE/MGD group.

The OSDI score that represents the ocular symptom did not differ among groups, except the normal group. The lid margin abnormality score was higher in the MGD and mixed groups compared to the normal group (*p* = .031, *p* = .012, respectively) and the ADDE group (*p* = .311, *p* = .145, respectively). The cornea and conjunctiva stained more prominently in the ADDE group compared to the MGD group (*p* < .001). The TBUT did not differ significantly among the ADDE, MGD, and mixed groups (all *p* > .05). The MGD group and the mixed group showed significantly higher meiboscores compared to the ADDE group (*p* = .004, *p* = .002, respectively). The LLT revealed similar results, where the MGD group and the mixed group had significantly lower LLTs than the ADDE group (*p* = .036, *p* = .009, respectively). The Schirmer test value was significantly lower in the ADDE group and the mixed group compared to the MGD group (*p* < .001, *p* < .001, respectively). It is noteworthy that the Schirmer test value was the highest in the MGD group, which was higher than in the normal group. A significantly higher MGD severity score was found in the MGD group and the mixed group when measured against the ADDE group (*p* = .001, *p* < .001, respectively).

**Figure 1.**
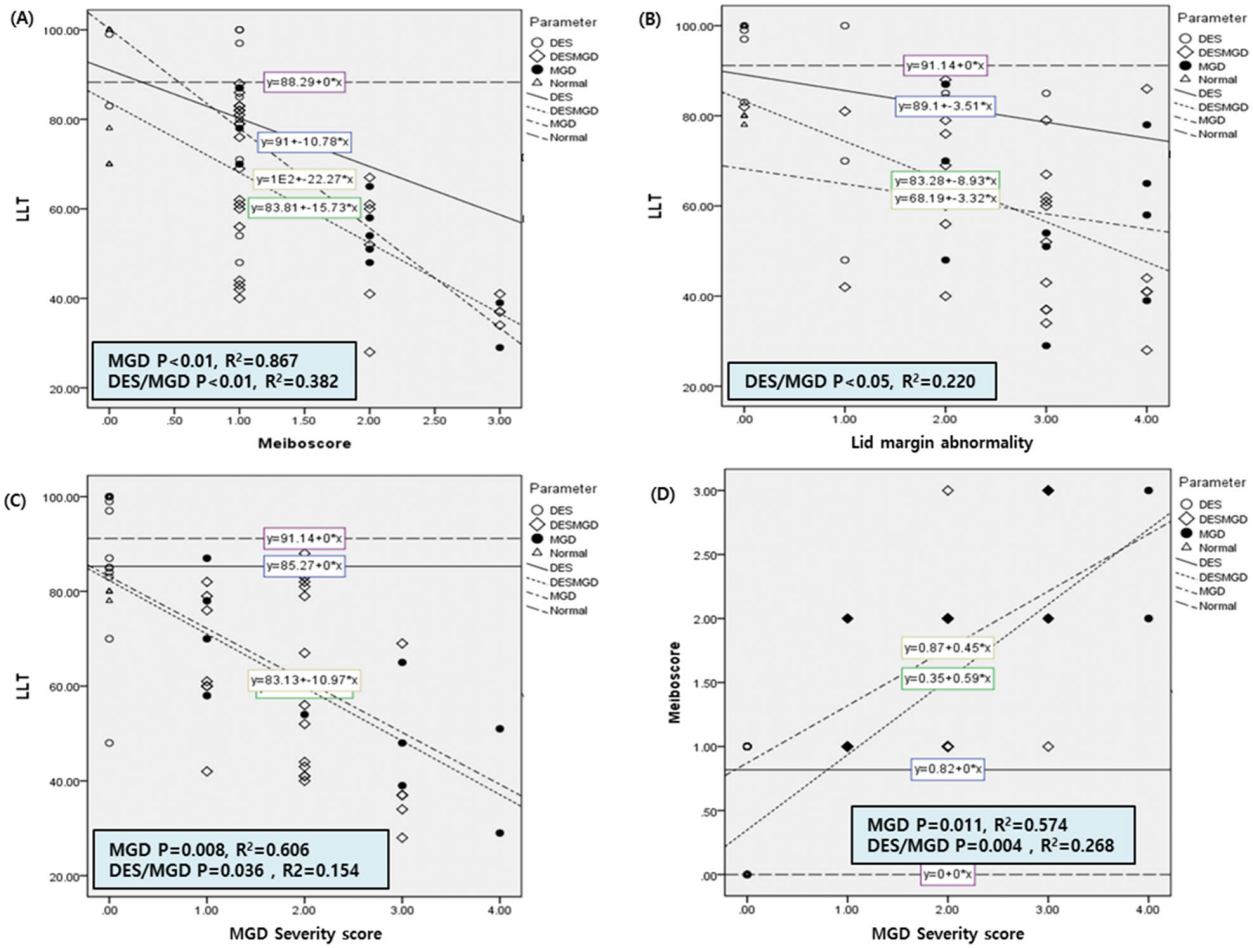
Scatterplots showing the correlations between lipid layer thickness (LLT) and Meibomian gland dysfunction (MGD) parameters. The triangles and the widely dotted lines represent the normal control group. The empty circles and the straight lines represent the aqueous deficient dry eye (ADDE) group. The filled circles and the alternatively dotted lines represent the MGD group. Finally, the diamonds and the narrowly dotted lines represent the Mixed group. (A) Correlation between LLT and meiboscore, which represents a Meibomian gland dropout grade of the lower lid. (B) Correlation between LLT and lid margin abnormality. (C) Correlation between LLT and MGD severity score. (D) Correlation between meiboscore and MGD severity score.

The scatterplot and regression curve for LLT versus the meiboscore in each group revealed a significant correlation between the two parameters in the MGD group and the mixed group (*p* < .01, *R*^2^=0.867 and *p* < .01, *R*^2^=0.382, respectively) (Figure 1(A)). The LLT seemed to negatively correlate with lid margin abnormality in the mixed group (*p* < .05, *R*^2^=0.220) (Figure 1(B)). A significant correlation was also found between LLT and MGD severity score in the MGD group and the mixed group (*p* = .008, *R*^2^=0.606 and *p* = .036, *R*^2^=0.154, respectively) (Figure 1(C)). There was a significant correlation between meiboscore and MGD severity score in the MGD group and the mixed group (*p* = .011, *R*^2^=0.574 and *p* = .004, *R*^2^=0.268, respectively) (Figure 1(D)).

**Figure 2.**
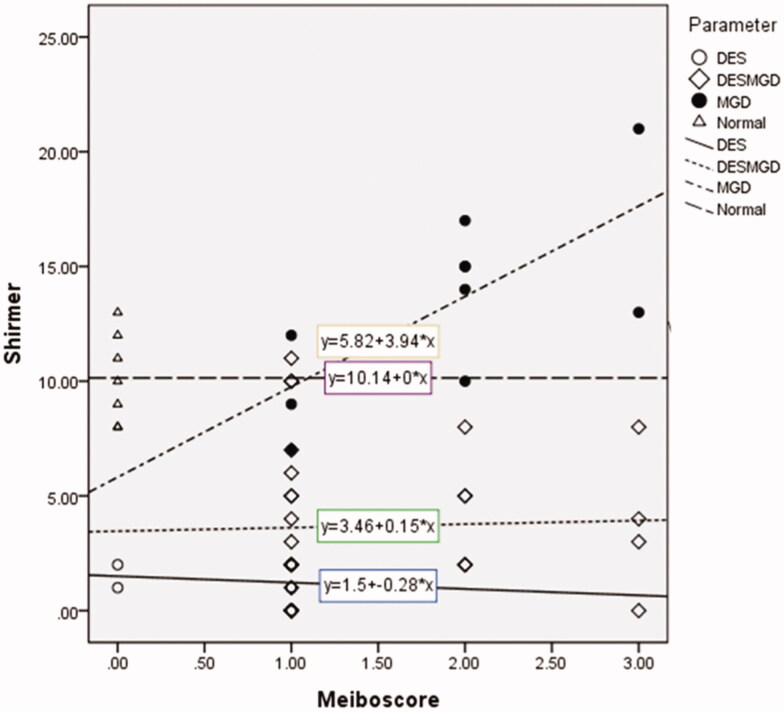
Scatterplot showing the correlation between the Schirmer test value and the meiboscore. The triangles and the widely dotted lines represent the normal control group. The empty circles and the straight lines represent the aqueous deficient dry eye (ADDE) group. The filled circles and the alternatively dotted lines represent the Meibomian gland dysfunction (MGD) group. Finally, the diamonds and the narrowly dotted lines represent the mixed group.

However, significant correlation between Schirmer test value and meiboscore was definite only in the MGD group (*p* = .021, *R*^2^=0.506) (Figure 2).

## Discussion

In the present study, we analyzed the clinical effects of the ocular surface and Meibomian gland parameters on tear film stability among individuals with MGD. MGD showed significantly lower LLT, while the Schirmer test value was significantly higher compared to the ADDE patients. Low LLT reflected the severity of Meibomian gland abnormality, which was indicated by the meiboscore. There was a meaningful correlation between Schirmer test value and meiboscore in the MGD group. Our study not only confirmed the results of Arita et al. [6] using a larger number of patients with dry eye and reported additional parameters related to the ocular surface, but also suggests obstructive MGD may be more prevalent than hypersecretory MGD.

In a recent review, a double vicious circle combining the related pathological mechanisms underlying DES and MGD was proposed. The dysfunction of the Meibomian gland, acted as not only the entry point into both the DES and MGD loops, but also connected the two vicious circles [16]. The Meibomian gland secretes the majority of lipids that comprises the lipid layer of the tear film and receives both parasympathetic and sympathetic innervation to regulate tear production with the lacrimal gland [17]. Therefore, Meibomian gland dysfunction may affect tear film stability and tear osmolarity, accounting for a major portion of the pathophysiology of DES [18].

In this study, the Schirmer test value and meiboscore data were collected as measures of aqueous tear production and Meibomian gland abnormality, respectively. Both tests are thought to be very reliable and are the most commonly performed tests in clinical practice. Arita et al. concluded that Schirmer test value and meiboscore were highly effective for differentiation of patients with MGD from those with non-Sjögren syndrome aqueous deficiency dry eye [19]. In addition, we evaluated LLT with the LipiView interferometer, which is capable of delivering quantitative data of the tear film oily layer. A number of studies have shown that the lipid layer is related to number and function of Meibomian glands [20–22]. LLT is negatively correlated with Meibomian gland loss in patients with MGD, which makes this parameter a helpful tool in diagnosis of MGD.

Experimental and clinical studies have suggested that there may be a compensatory reaction of the tear film to a damaged Meibomian gland [23,24]. In a rabbit model, an eye with surgically obstructed Meibomian gland orifices exhibited increased Schirmer test values and tear osmolarity compared to the control eye [25]. Shimazaki et al. demonstrated that patients with MGD had a greater degree of ocular surface damage, which was reflected by higher staining with fluorescein or rose Bengal, and higher Schirmer test values than patients without MGD [26]. A recent study found that tear secretion in MGD patients was increased markedly according to extent of Meibomian gland loss (meiboscore) [6]. The authors interpreted these results to mean that discomfort due to increased friction during blinking in MGD patients whose lipid layer is decreased led to compensatory reflex tearing. This is consistent with our results that MGD patients with higher meiboscore exhibited higher Schirmer test value. Furthermore, meiboscore along with other MGD parameters, such as lid margin abnormality and MGD severity score, showed a negative correlation with LLT, which suggests that tear fluid secretion is increased to compensate for reduced Meibomian gland function and the subsequent tear film instability caused by deficiency of the lipid layer.

Interestingly, our data showed no significant difference in TBUT between the ADDE and MGD patients. This is in contrast with other studies, which showed shorter TBUT in ADDE patients compared to MGD patients [6,22,27]. However, in correlation analysis, higher meiboscore was correlated to shorter TBUT. In addition, the thinner the lipid layer was, the shorter the time it took for the tear film to breakup. A possible explanation of this discrepancy is that tear film is not only affected by tear film stability but also by blinking, palpebral fissure size, ocular surface state, and the mucin and aqueous layers.

In this study, the LLT negatively correlated with the meiboscore and the MGD severity score in the MGD group (*p* < .01, *R*^2^ = 0.867 and *p* < .008, *R*^2^ = 0.606, respectively). LLT was not increased but decreased, according to the increased meiboscore and MGD severity score in the MGD group. And there was no correlation between the LLT with the meiboscore and the MGD severity score in the ADDE and the MGD group (mixed group) (*p* > .05 respectively). According to our study, we hypothesized that obstructive MGD maybe more prevalent than hypersecretory MGD in the MGD group.

The limitation of this study is only including data of lower Meibomian glands. The secretory capacity of the upper Meibomian glands may be larger than that of the lower Meibomian glands [28]. Although we only assessed the lower eyelid, according to previous studies, upper Meibomian gland loss was correlated with lower Meibomian gland loss, and both were correlated with lipid layer thickness in obstructive MGD [22, 28]. Therefore, a single evaluation of the lower lid may be enough for measurement of MGD. However, the evaluation of both upper and lower meiboscore would be more precise to determine the function of Meibomian gland. Further investigations dealing with tear osmolarity; the condition of the mucin layer and inflammatory factors such as IL-1ß, IL-6, IL-12, and TNF-α; and blinking are required to confirm the components that maintain tear film homeostasis. This thesis was mainly about obstructive MGD, and further studies about prevalence between hypo and hypersecretory MGD are necessary.

In conclusion, we described the first study using an interferometer to show that tear fluid secretion was increased in patients with MGD compared to ADDE patients, and that an actual correlation existed between tear fluid secretion and lipid layer thickness. Our results suggest that tear film instability through decreased lipid layer thickness, caused by Meibomian gland dysfunction, further stimulates the ocular surface to cause reflex tear secretion. Lipid layer thickness is more decreased in MGD patients compared to ADDE patients. Therefore, obstructive MGD may be more prevalent than hypersecretory MGD.

## Data Availability

The datasets used and/or analyzed during the current study available from the corresponding author on reasonable request.
